# Opioid-sparing effects of ultrasound-guided erector spinae plane block for video-assisted thoracoscopic surgery: a randomized controlled study

**DOI:** 10.1186/s13741-024-00413-8

**Published:** 2024-06-07

**Authors:** Huan Xu, Wei Wu, Xue Chen, Wenxin He, Hong Shi

**Affiliations:** 1 Department of Anesthesiology, Shanghai Pulmonary Hospital, School of Medicine, Tongji University, Shanghai, 200433 China; 2 Department of Thoracic Surgery, Shanghai Pulmonary Hospital, School of Medicine, Tongji University, Shanghai, 200433 China

**Keywords:** Erector spinae plane block, Video-assisted thoracoscopic surgery, Analgesia, Opioid, Pain

## Abstract

**Background:**

The erector spinae plane block (ESPB) is a new analgesic method used in thoracic surgery. However, few studies have characterized their effects on perioperative opioid consumption. We aimed to evaluate the effects of ESPB on perioperative opioid consumption in patients who underwent video-assisted thoracoscopic surgery (VATS).

**Methods:**

This was a randomized, observer-blinded clinical trial at a single-centre academic hospital. Eighty patients were scheduled for thoracoscopic segmentectomy or lobectomy by VATS for lung cancer. Forty participants were randomly assigned to ESPB or control group. All patients received intravenous patient-controlled postoperative analgesia. Perioperative opioid consumption, visual analogue scale (VAS) scores, and adverse events were recorded.

**Results:**

Intraoperative and postoperative opioid consumption and static/dynamic VAS scores were significantly lower in the early hours after VATS in the ESPB group (*p* < 0.05) than the control group. No significant differences were observed in adverse effects between the two groups.

**Conclusions:**

ESPB reduced intraoperative opioid consumption and early postoperative pain in patients undergoing VATS. Our findings support the view that ESPB is a safe and highly effective option for regional analgesia for VATS.

**Trial registration:**

http://www.chictr.org.cn, ChiCTR1800019335.

## Background

Video-assisted thoracoscopic surgery (VATS) is a minimally invasive technique (Klapper and D’Amico 2015). VATS improved postoperative respiratory function and shortened the length of hospital stay (Kaseda et al. 2000, Flores et al. 2008). Management of the associated moderate-to-severe postoperative pain (Steinthorsdottir et al. 2014) is critical for enhanced recovery after surgery. Opioids are the most commonly used analgesics during this period. However, due to their adverse effects (Daly and Myles 2009), multimodal analgesia methods are preferred. The erector spinae plane block (ESPB) is an emerging regional anaesthetic technique used for surgeries and acute and chronic pain management (Tulgar et al. 2019). ESPB, first used in 2016 (Forero et al. 2016), is simple and facilitates minimal or no sedation in the preoperative holding area. Various ongoing trials and prospective studies are focused on ESPB; however, few studies have characterized its effects on perioperative opioid consumption. Therefore, this study aimed to evaluate its effects on perioperative opioid consumption during VATS.

## Methods

### Ethics approval and informed consent

This study was approved by the Medical Ethics Committee of Shanghai Pulmonary Hospital and registered in Chinese Clinical Trial Registry (ChiCTR1800019335). All participants provided written informed consent. At the same time, this study conforms to the principles outlined in the Declaration of Helsinki.

### Study participants

We enrolled patients with the American Society of Anaesthesiologists physical status I or II, aged 20–80 years, scheduled for thoracoscopic segmentectomy or lobectomy by VATS for lung cancer. We excluded patients with a history of drug abuse, chronic pain, psychological disorders, preoperative chest pain for > 3 months, allergies to local anaesthetics, and difficulty understanding the study protocol.

The patients were randomly allocated using the random number table method to receive a single-shot, ESPB with either a 25-mL mixture dose of 1% lidocaine and epinephrine (1:200,000) combined with 0.5 µg/mL sufentanil (ESPB group) or no block (control group) before general anaesthesia. The treatment allocations were sealed in opaque envelopes and opened the day before surgery by a researcher not involved in the trial. The procedure was performed in the preoperative regional room before administering anaesthesia. Due to the inherent nature of our study, blinding the anaesthesiologists is not feasible. However, the surgeons, postoperative medical team, and evaluators will not be informed of the participants’ group assignment.

### Application of ESPB

Ultrasound-guided ESPB was performed in the lateral decubitus position under standardized monitoring before the induction of general anaesthesia. A high-frequency linear-array ultrasound probe was placed in a sterile sheath. Ultrasound-guided ESPB was administered at the T5 vertebral level. The probe was placed longitudinally, 2–3 cm lateral to the midline. The trapezius, rhomboid, and erector ridge muscles and tip of the T5 transverse process were clearly visible from the top down. A 50-mm 22-g needle was inserted into the interfascial area between the erector spinae muscle and transverse process of the vertebra using an in-plane technique. After the correct location was confirmed by hydrodissection of the interfascial plane with 2 mL of normal saline, a 25-mL mixture dose of 1% lidocaine and epinephrine (1:200,000) combined with 0.5 µg/mL sufentanil was injected. Successful block was then confirmed based on loss of cold sensation on wiping the area with an alcohol cotton swab three times.

### General anaesthesia

After the block procedure, patients were transferred to the operating room. The patients were monitored using an electrocardiogram (ECG), noninvasive blood pressure measurement, bispectral index (BIS), and pulse oximetry saturation (SPO_2_). Following the placement of a peripheral intravenous catheter, 2-mg IV midazolam was administered for sedation. Propofol (1.5–2.5 mg/kg), sufentanil (0.4–0.5 µg/kg), and rocuronium bromide (0.5–0.6 mg/kg) were used for anaesthesia induction. Tracheal intubation was performed using a left-sided, 35–39 French double-lumen tube. Tube position was corrected using fibreoptic bronchoscopy. Anaesthesia was maintained with propofol and targeted at a BIS of 40–60. During the surgical procedure, 5–10 μg of sufentanil was administered intravenously to both groups for maintaining systolic blood pressure changes within 20% of the baseline. This dose was repeated every 20 min until the blood pressure returned to the required limits. Then, 4-mg IV ondansetron was administered to prevent postoperative nausea and vomiting. At the end of the surgery, IV neostigmine (0.02 mg/kg) and atropine (0.01 mg/kg) were available for reversal of muscle relaxant, if necessary. The patients recovered in the post-anaesthesia care unit (PACU) for 1 h.

### Postoperative analgesia management

Postoperative pain management was performed using a standardized protocol for all patients. An intravenous patient-controlled analgesia (IVPCA) device was connected to each patient at the PACU and was maintained postoperatively using the following protocol: 1 mL/h (1 µg/mL sufentanil + 1 mg/mL flurbiprofen axetil + 1 mg/mL tropisetron hydrochloride) basal infusion with a 2-mL bolus dose, a 15-min lockout time, and a maximum limit of 10 mL/h. Static (at rest) and dynamic (with movement while coughing) pain scores were evaluated using VAS scores (0 = no pain, 10 = most severe pain). VAS scores were recorded at 1, 6, and 24 h, 1 week, and 1 month postoperatively. Additionally, 25 mg of IV meperidine is administered as a rescue analgesic on demand (VAS score, > 4). Adverse effects of postoperative opioid consumption such as nausea, vomiting, dizziness, and itching were also recorded.

### Outcome

The primary outcome was intraoperative sufentanil consumption. The secondary outcomes were sufentanil consumption at 24 h postoperatively and VAS scores at rest and with movement at 1, 6, and 24 h, 1 week, and 1 month postoperatively. The mean arterial pressure (MAP), heart rate (HR), and postoperative opioid-related adverse events were recorded. Patient satisfaction with the effectiveness of analgesia during the initial 48 h postoperatively assessed using a 5-point Likert scale, ranging from “highly unsatisfactory” to “highly satisfactory”.

### Statistical analysis

Sample size calculation was conducted using PASS software version 15 for two-sample *t*-test. Based on preliminary data from our institution involving 10 patients, the mean (standard deviation) of intraoperative sufentanil consumption was estimated to be 48.9 (10.1) in the ESPB group and 55.6 (6.6) in the control group. With a statistical power of 90% and a significance level (α) of 0.05, it was determined that a minimum of 36 patients per group would be necessary to detect a significant difference. Accounting for a 10% dropout rate, a total sample size of 80 patients was planned, with 40 patients allocated to each group.

Data were analysed using the IBM SPSS Statistics software version 25.0 (SPSS Inc., Chicago, IL, USA). Continuous variables were compared using the unpaired *t*-test or Mann–Whitney *U*-test, and categorical variables were compared using the chi-square test or Fisher’s exact test. All data are summarized as mean (SD) or median (25–75% range), as appropriate. Multiple comparisons of opioid consumption over different periods were performed using the Bonferroni method. *P* < 0.05 was considered statistically significant.

## Results

**Fig. 1:**
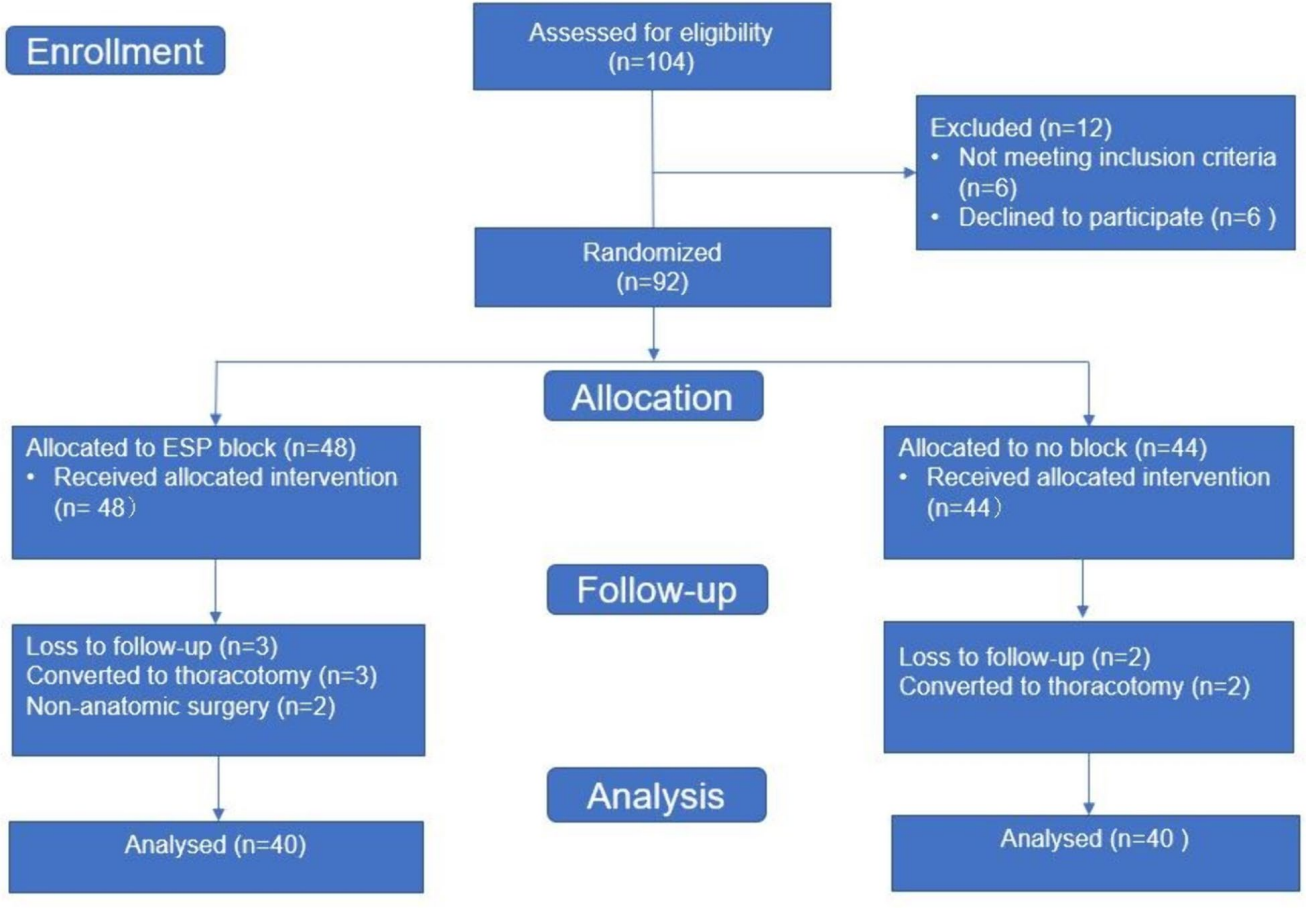
Flowchart of the study

From January 2019 to January 2021, a total of 92 patients went randomization. After excluding 12 patients, the final analysis included 80 patients. In each group, 40 participants were randomly assigned, as presented in the Consolidated Standards of Reporting Trials (CONSORT) flow chart (Fig. 1). The demographic data and surgical duration were comparable between the two groups (Table 1). No significant differences were observed in the demographic data, surgery duration, and anaesthesia between the two groups (*p* > 0.05).

**Table 1:** Characteristics of the participants and their thoracoscopic operations [mean (standard deviation)]

	**ESPB group (*n* = 40)**	**Control group (*n* = 40)**
Sex: male/female	22/18	18/22
Age: year	60.8 (8.1)	58.4 (10.9)
Height: cm	162.83 (8.3)	162 (8.4)
Weight: kg	66.15 (10.2)	63.38 (9.0)
BMI: kg/m^2^	24.88 (2.9)	24.10 (1.6)
ASA: I/II	16/24	19/21
Intraoperative		
Operation: RUL/RML/RLL/LUL/LLL	15/3/4/11/7	13/6/7/9/5
Port number: 1/2	36/4	36/4
Duration: min	113.03 (37.1)	104 (41.2)
Estimated blood loss: mL	59.5 (33.4)	52.75 (39.9)
Urine: mL	281.25 (156.4)	233.75 (86.5)
Duration of anaesthesia: min	132.5 (37.5)	132.9 (43.4)

*BMI* Body mass index, *ESPB* Erector spinae plane block, *RUL* Right upper lobe, *RML* Right middle lobe, *RLL* Right lower lobe, *LUL* Left upper lobe, *LLL* Left lower lobe

### Primary endpoint

The intraoperative opioid consumption (52.63 ± 9.57 vs 58.63 ± 6.10 µg) in the ESPB group was significantly lower than in the control group (*p* < 0.05) (Table 2).

**Table 2:** Comparison of postoperative and intraoperative sufentanil consumption (mean ± standard deviation)

	**ESPB group (*n* = 40), µg**	**Control group (*n* = 40), µg**	***p*-value**
Intraoperative sufentanil consumption	52.63 ± 9.57	58.63 ± 6.10	0.001
Postoperative sufentanil consumption, time after surgery			
0–1 h	1.10 ± 0.44	1.90 ± 1.06	< 0.001
1–6 h	4.75 ± 2.12	6.10 ± 2.81	0.018
6–24 h	18.98 ± 5.17	18.98 ± 5.15	1.00
Total postoperative sufentanil consumption, 0–24 h	24.83 ± 4.30	26.98 ± 6.43	0.083

*ESPB* Erector spinae plane block

### Secondary endpoint

**Fig. 2:**
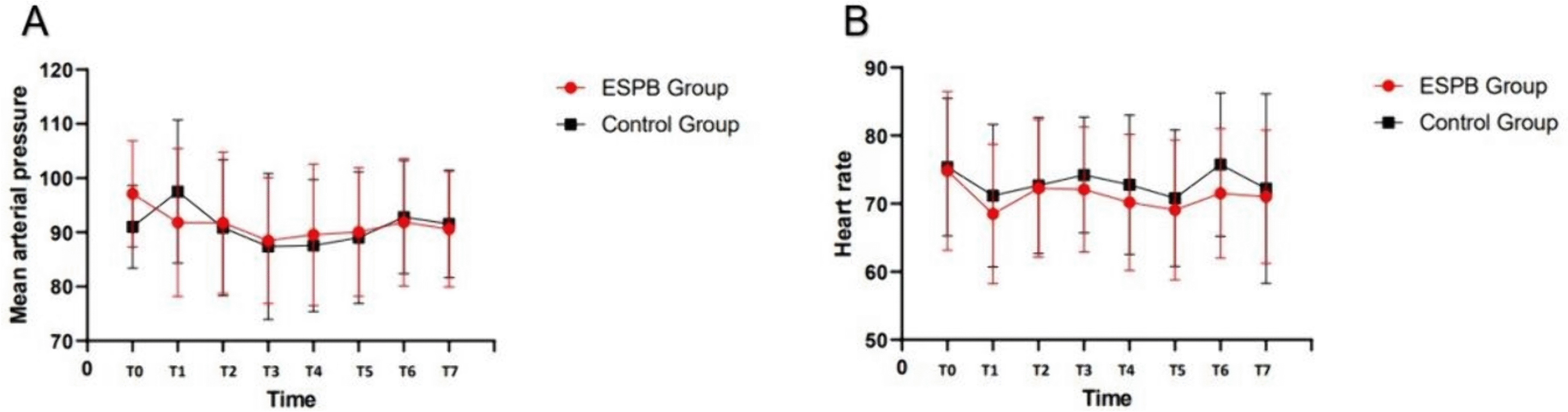
Changes in mean arterial pressure (MAP) and heart rate (HR) between groups over time. T0, baseline; T1, at the time of skin incision; T2, 10 min after surgery began; T3, 20 min after surgery began; T4, 40 min after surgery began; T5, 60 min after surgery began; T6, at the time of entering PACU; T7, 1 h of staying in PACU

The postoperative opioid consumption at 0–1 h (1.10 ± 0.44 vs 1.90 ± 1.06 µg) and 1–6 h (4.75 ± 2.12 vs 6.10 ± 2.81 µg) in the ESPB group was significantly lower than in the control group (*p* < 0.05). At 6–24 h, postoperative opioid consumption was similar between the two groups (18.98 ± 5.17 vs 18.98 ± 5.15 µg, *p* > 0.05) (Table 2). No significant difference was observed in the total postoperative 24-h opioid consumption between the two groups (24.83 ± 4.30 µg vs 26.98 ± 6.43 µg, *p* > 0.05) (Table 2). The VAS pain scores at rest and with movement were significantly lower in the ESPB group than in the control group 1 h after surgery (*p* < 0.001). However, no significant differences were observed between the static/dynamic VAS scores of the two groups at 6 and 24 h, 1 week, and 1 month, post-surgery (*p* > 0.05) (Table 3). MAP and HR during surgery and after 1 h in the PACU were similar between the groups (*p* > 0.05) (Fig. 2). Although the ESPB group had a decreasing trend in postoperative nausea and vomiting, no significant differences were observed in the symptoms between the two groups. Moreover, no differences were observed in the other postoperative adverse effects and patient satisfaction scores between the two groups (Tables 4 and 5).

**Table 3:** Assessment of postoperative pain scores [median (IQR)]

Time		Outcome	ESPB group (*n* = 40)	Control group (*n* = 40)	*p*-value
**1 h**	VAS				
		At rest	0 (1)	1 (1)	< 0.001
		With movement	1 (1)	3 (1)	< 0.001
**6 h**	VAS				
		At rest	3 (1)	3 (2)	0.968
		With movement	4.5 (1)	4 (1)	0.383
**24 h**	VAS				
		At rest	2 (2)	3 (2)	0.461
		With movement	4 (3)	4 (2)	0.786
**1 week**	VAS				
		At rest	1 (1)	1 (1)	0.715
		With movement	2 (1)	2 (1)	0.747
**1 month**	VAS				
		At rest	0 (0)	0 (0)	0.130
		With movement	1 (1)	0 (1)	0.057

*VAS* Visual analogue scale, *IQR* Interquartile range

**Table 4:** Comparison of incidence of postoperative opioid-related adverse effects

Time	Outcome	ESPB group (*n* = 40)	Control group (*n* = 40)	*p*-value
**6 h**	Breathing depression	0	0	0
	Nausea	8 (20%)	16 (40%)	0.051
	Vomiting	6 (15%)	12 (30%)	0.108
	Dizzy	5 (12.5%)	8 (20%)	0.363
	Intestinal exhaust	2 (5%)	3 (7.5%)	0.644
	Itching	0	0	0
**24 h**	Breathing depression	0	0	0
	Nausea	8 (20%)	14 (35%)	0.133
	Vomiting	6 (15%)	13 (32.5%)	0.066
	Dizzy	10 (25%)	8 (20%)	0.592
	Intestinal exhaust	34 (85%)	32 (80%)	0.556
	Itching	0	0	0

*ESPB* Erector spinae plane block

**Table 5:** Patients’ satisfaction scores for postoperative pain management

Satisfaction	ESPB group (*n* = 40)	Control group (*n* = 40)	*p*-value
Extremely satisfactory	7	15	0.079
Satisfactory	23	20	
Neither satisfactory nor unsatisfactory	10	4	
Unsatisfactory	0	1	
Extremely unsatisfactory	0	0	

*ESPB* Erector spinae plane block

## Discussion

The results of this study revealed that intraoperative and postoperative opioid consumption at 0–1 h and 1–6 h in the ESPB group was significantly lower than that in the control group. Simultaneously, preoperative ultrasound-guided ESPB resulted in lower static/dynamic VAS pain scores in the first 1 h after thoracoscopic lung surgery compared to the control. The ESP block provides more effective postoperative pain control and results in less opioid consumption, especially during the stay in the PACU. The postoperative opioid-related adverse effects did not differ between the two groups. Although an increasing trend in postoperative nausea and vomiting was observed in the control group, the difference between the two groups was not statistically significant.

VATS is a standard surgical procedure for lung cancer and a well-established routine at our hospital. Peripheral regional anaesthesia (RA) techniques are commonly used in VATS or thoracotomy even in the absence of accurate indications regarding their effectiveness on postoperative pain management. RA is a useful choice in thoracic surgery. However, it is still impossible to determine the most appropriate block in the individual surgical settings to be performed due to RCTs paucity (Balzani et al. 2023). As preoperative regional analgesia may reduce the effects of neuromodulation and improve postoperative pain control (Katz et al. 2003), we administered an ESP block before surgery. The mean duration of surgery was 113.03 ± 37.1 min in the ESPB group; therefore, we assume that the main effect of the block was on intraoperative opioid consumption. Thus, the intraoperative sufentanil consumption was 52.63 ± 9.57 µg in the ESPB group, while it was 58.63 ± 6.10 µg in the control group. Opioids have been reported to suppress immune function, affecting tumour metastasis and recurrence (Brack et al. 2011). During thoracoscopic surgery, we selected an ESP block combined with intravenous anaesthesia to decrease the dose of opioids and potentially reduce immune function inhibition. Therefore, if ESPB analgesia is selected, patients receiving the block will have better recovery. According to new research in the various regional analgesic techniques used in thoracic surgery, evidence suggests that ESPB might be the most effective and safest method for enhancing pain relief after thoracic surgery, shortening the length of hospital stay and reducing the incidence of postoperative complications (Li et al. 2023).

Previous studies have reported different effective ESPB volumes and concentrations for thoracoscopic surgery. A previous study reported that ESPB provided effective analgesia for 6 h postoperatively (Kendall et al. 2020). However, our study demonstrated different durations of postoperative analgesia. In our study, preoperative ESPB with lidocaine resulted in lower VAS pain scores 1 h after thoracoscopic lung surgery. The postoperative analgesic effect of the block may have reduced based on the duration of the surgery. To counter this effect, continuous infusion using a catheter may be preferred. Any systemic effect may be less significant in single-shot ESP blocks than in continuous blocks and probably cannot explain the prolonged postoperative analgesia reported in clinical studies. However, the continuous block may be more invasive and may increase complications than a single-shot injection. Therefore, performing the ESP block with different adjuvant agents (such as dexmedetomidine) should be explored further.

Residual anaesthetics and postoperative analgesics may cause adverse effects including nausea, vomiting, dizziness, and respiratory depression. The results revealed that the incidence of adverse effects after surgery did not differ between the two groups. Previous studies demonstrated that the ESP block resulted in a significant reduction in opioid consumption and can reduce postoperative nausea and vomiting caused by opioids (Kendall et al. 2020; Cui et al. 2022). This discrepancy could be because of different reasons. Firstly, we did not use ESPB as a postoperative analgesic method; therefore, its benefits were not completely represented. Secondly, enhanced recovery after the VATS protocols can prevent factors that delay postoperative recovery and cause complications. Similar results have been reported in other studies (Ljungqvist et al. 2017).

The present study had several limitations. First, we did not perform the block with placebo injectate or a sham procedure, which increased the risk of performance and assessment biases. Second, the ESP block should be performed after VATS as it can better demonstrate the differences between the two groups. Third, the utilization of opioid medications during surgery in our practice is informed by the clinical experience and established protocols within our institution, albeit acknowledging potential limitations. Another limitation is that our study did not assess the incidence of postoperative complications including postoperative pneumonia, surgical site infection, and acute kidney injury. Previous studies have reported that regional anaesthesia may reduce the incidence of these complications (Finnerty et al. 2020). Moreover, a block catheter could be used for continuous infusion to provide postoperative pain control in future studies.

## Conclusion

ESPB reduced intraoperative sufentanil consumption and the first hour sufentanil consumption after surgery. The early postoperative pain in patients undergoing VATS was significantly lower in the ESPB group than in the control group. Our findings support the view that ESPB is a safe and effective option for regional analgesia for VATS.

## Data Availability

The datasets generated during and/or analysed during the current study are available from the corresponding author on reasonable request.

## Abbreviations

ESPB: Erector spinae plane block
VATS: Video-assisted thoracoscopic surgery
VAS: Visual analogue scale
ECG: Electrocardiogram
BIS: Bispectral index
SPO_2_: Pulse oximetry saturation
PACU: Post-anaesthesia care unit
IVPCA: Intravenous patient-controlled analgesia
MAP: Mean arterial pressure
HR: Heart rate
CONSORT: Consolidated Standards of Reporting Trials
